# Critical Issues in *BDNF* Val66Met Genetic Studies of Neuropsychiatric Disorders

**DOI:** 10.3389/fnmol.2018.00156

**Published:** 2018-05-15

**Authors:** Shih-Jen Tsai

**Affiliations:** ^1^Department of Psychiatry, Taipei Veterans General Hospital, Taipei, Taiwan; ^2^School of Medicine, National Yang-Ming University, Taipei, Taiwan; ^3^Institute of Brain Science, National Yang-Ming University, Taipei, Taiwan

**Keywords:** brain-derived neurotrophic factor, Val66Met polymorphism, transgenic mice, genetic study, age, sex, environmental factors, ethnicity

## Abstract

Neurotrophins have been implicated in the pathophysiology of many neuropsychiatric diseases. Brain-derived neurotrophic factor (BDNF) is the most abundant and widely distributed neurotrophin in the brain. Its Val66Met polymorphism (refSNP Cluster Report: rs6265) is a common and functional single-nucleotide polymorphism (SNP) affecting the activity-dependent release of BDNF. *BDNF* Val66Met transgenic mice have been generated, which may provide further insight into the functional impact of this polymorphism in the brain. Considering the important role of BDNF in brain function, more than 1,100 genetic studies have investigated this polymorphism in the past 15 years. Although these studies have reported some encouraging positive findings initially, most of the findings cannot be replicated in following studies. These inconsistencies in *BDNF* Val66Met genetic studies may be attributed to many factors such as age, sex, environmental factors, ethnicity, genetic model used for analysis, and gene–gene interaction, which are discussed in this review. We also discuss the results of recent studies that have reported the novel functions of this polymorphism. Because many *BDNF* polymorphisms and non-genetic factors have been implicated in the complex traits of neuropsychiatric diseases, the conventional genetic association-based method is limited to address these complex interactions. Future studies should apply data mining and machine learning techniques to determine the genetic role of *BDNF* in neuropsychiatric diseases.

## Introduction

Brain-derived neurotrophic factor (BDNF), a major member of the neurotrophin family, is widely expressed in the mammalian brain (Hofer et al., 1990). The highest level of BDNF is found in the hippocampus and the cerebral cortex, which are regions of the brain that are involved in many neuropsychiatric diseases (Hofer et al., 1990). BDNF is critical to the growth, survival, and differentiation of the developing nervous system through its binding to a high affinity tyrosine kinase receptor B (TrkB) and/or the p75 neurotrophin receptor. Mutant mice lacking BDNF exhibit developmental brain abnormalities and die soon after birth (Ernfors et al., 1994). In addition, BDNF can modulate synaptic transmission and activity-dependent plasticity, and it can promote long-term potentiation (LTP) (Xu et al., 2000; Bramham and Messaoudi, 2005).

**FIGURE 1:**
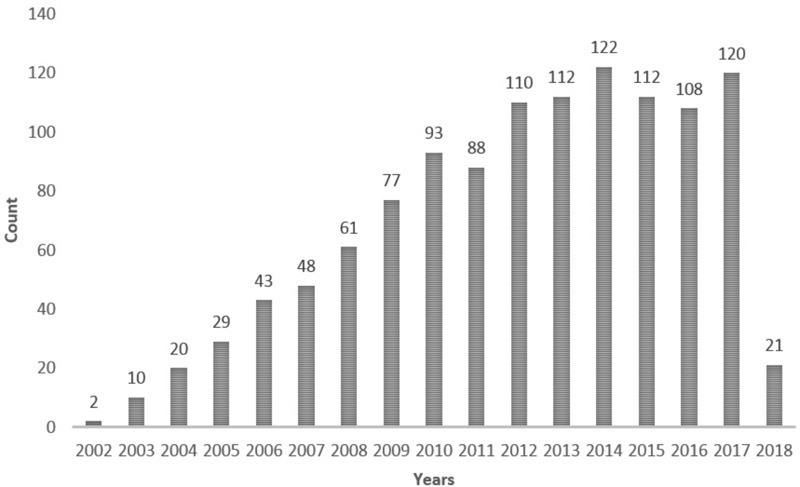
A search for reports on the *BDNF* Val66Met polymorphism with the keywords “(bdnf val66met) OR rs6265 OR (bdnf g196a) OR (bdnf 196g/a) OR (bdnf 196a/g) OR (bdnf 196 a/g)” performed in the PubMed database up to February 14, 2018.

The human *BDNF* gene is located on chromosome 11p13 and has 11 exons and 9 functional promoters that are brain region- and tissue-specific (Pruunsild et al., 2007). In this gene, a non-synonymous polymorphism (refSNP Cluster Report: rs6265; also called Val66Met or G196A polymorphism) is common; this polymorphism causes a valine (Val) to methionine (Met) change at position 66 of the proBDNF protein. The replacement of Val by Met impairs the neuronal activity-dependent secretion of BDNF (Egan et al., 2003). The first two genetic studies investigating the *BDNF* Val66Met polymorphism were published in 2002 (Momose et al., 2002; Ventriglia et al., 2002). Considering the important role of BDNF in the brain, over the past 15 years, many genetic studies have investigated the effects of this *BDNF* polymorphism on brain function and behavior in health, as well as in diseases, particularly neuropsychiatric diseases (Hong et al., 2011; Notaras et al., 2015b) (**Table 1**). A search with the keywords “(bdnf val66met) OR rs6265 OR (bdnf g196a) OR (bdnf 196g/a) OR (bdnf 196a/g) OR (bdnf 196 a/g)” performed in the PubMed database up to February 14, 2018 found 1,176 reports on this polymorphism (**Figure 1**). Although many reports have demonstrated the possible genetic effects of this *BDNF* polymorphism in diseases or brain function, other reports have failed to replicate the findings. The inconsistent findings of *BDNF* Val66Met genetic studies may result from many factors such as age, sex, environmental factors, ethnicity, genetic model used for analysis, and gene–gene interaction. In this review, we discuss these issues in genetic studies of the *BDNF* Val66Met polymorphism. We also discuss some findings for the novel function of this polymorphism.

**Table 1:** Meta-analyses of studies of the *BDNF* Val66Met polymorphism in neuropsychiatric diseases.

		Number of
Disease/phenotype	Studies	studies	Participants	Result
Major depressive disorder	Verhagen et al., 2010	14	2,812 cases; 10,843 controls	Met increased risk for depression in men but not in women.
	Pei et al., 2012	5	523 cases; 1,220 controls	Met increased risk for geriatric depression.
	Gyekis et al., 2013	26	4,582 cases; 12,995 controls	Lack of association.
	Hosang et al., 2014	22	14,233 participants	Val66Met polymorphism significantly moderated the relationship between life stress and depression.
	Zhao M. et al., 2017	31	21,060 participants	Life stress interacted with the Met in depression risk.

Response to antidepressant	Zou et al., 2010	8	1,115 cases	Val66Met heterozygous patients had a better response rate in comparison to Val homozygous patients, especially in Asian population.
	Yan et al., 2014	16		Met carriers had a better response rate than Val/Val carriers in Asians.

Suicide behaviors	Zai et al., 2012	12	1,202 cases; 2,150 controls	Met carriers and Met allele conferred risk for suicide.
	Gonzalez-Castro et al., 2017	23	4,532 cases; 5,364 controls	Met is the risk allele in Caucasian; Val is the risk allele in Asian.

Bipolar disorder	Kanazawa et al., 2007	11	3,143 cases; 6,347 controls	Lack of association.
	Gonzalez-Castro et al., 2015	22	9,349 cases; 7,437 controls	Lack of association.
	Wang et al., 2014	21	7,219 cases; 9,832 controls	Lack of association.

Schizophrenia	Zintzaras, 2007	9	1,404 cases; 1597 controls	Lack of association.
	Xu et al., 2007	11	3,032 cases; 4,080 controls	Lack of association.
	Naoe et al., 2007	8	2,059 cases; 2,765 controls	Lack of association.
	Gratacos et al., 2007	12	3,338 cases; 4,635 controls	Met/Met increased the risk of schizophrenia.
	Qian et al., 2007	16	2,991 cases; 3,962 controls	Lack of association.
	Kanazawa et al., 2007	13	2,955 cases; 4,035 controls	Lack of association.
	Kawashima et al., 2009	22	6,568 cases; 8,824 controls	Lack of association.
	Kheirollahi et al., 2016	39		Met/Met increased the risk of schizophrenia in Asian and European populations.
	Zhao et al., 2015	44	11,480 cases; 13,490 controls	Lack of association.

Response to antipsychotics	Cargnin et al., 2016	9	2,461 antipsychotic-treated patients	Lack of association.

Antipsychotic-induced tardive dyskinesia	Miura et al., 2014	6	1,740 antipsychotic-treated patients	Lack of association.

Generalized Anxiety Disorder	Frustaci et al., 2008	7	1,092 cases; 8,394 controls	Lack of association.

Neuroticism	Frustaci et al., 2008	5	1,633 participants	Met carriers had lower Neuroticism score.

Posttraumatic stress disorder (PTSD)	Wang, 2015	6	696 cases; 1,726 controls	Lack of association.
	Bruenig et al., 2016	9	1,066 cases; 2,559 were controls	Met carriers had increased risk of PTSD.

Panic disorder	Chen et al., 2017	6		A significant association in recessive model.

Obsessive-compulsive disorder	Wang et al., 2015	8	1,632 cases; 2,417 controls	Lack of association.

Attention-deficit hyperactivity disorder	Sanchez-Mora et al., 2010	4	1,445 adulthood patients; 2,247;controls	Lack of association.

Eating disorder	Gratacos et al., 2007	5	1,733cases; 1,811 controls	Met increased the risk of eating disorder.
	Brandys et al., 2013	9	2,767 cases; 3,322 controls	Lack of association.

Cognition	Kambeitz et al., 2012	32	5,922 participants	Met carriers performed worse than the Val homozygotes in memory.
	Mandelman and Grigorenko, 2012	23	7,095 participants	Lack of association.

Hippocampal volume	Hajek et al., 2012	7	399 participants	Met carriers had smaller hippocampal volumes than Val homozygotes.
	Harrisberger et al., 2014	27	5,298 participants	Met carriers had slightly smaller hippocampal volumes than Val homozygotes.
	Harrisberger et al., 2015	18	1,695 neuropsychiatric patients	Lack of association.

Alcohol dependence	Forero et al., 2015	9	2,553 cases; 2,709 controls	Lack of association.

Substance abuse	Gratacos et al., 2007	6	1,361 cases; 1,164 controls	Val homozygotes conferred risk for substance abuse.
	Haerian, 2013	20	4,665 cases; 4,754 controls	Val increased the risk of methamphetamine dependence in south Asian participants and the risk of heroin dependence in Chinese participants.

Adult-onset dystonia	Gomez-Garre et al., 2014	7	1,936 cases; 2,519 controls	Lack of association.

Migraine	Terrazzino et al., 2017	5	1,442 cases; 1,880 controls	Met increased the risk of migraine.
	Cai et al., 2017	4	1,598 cases; 1,585 controls	Met increased the risk of migraine.

Parkinson’s disease	Zintzaras and Hadjigeorgiou, 2005	6	1,419 cases; 1,406 controls.	Lack of association.
	Dai et al., 2013	13	3,333 cases; 3,418 controls	Lack of association.
	Mariani et al., 2015	15	3,754 cases; 4,026 controls	Lack of association.

Alzheimer’s disease (AD)	Fukumoto et al., 2010	16	4,711 cases; 4,537 controls	Met increased the risk of AD in women, but not in men.
	Lin et al., 2014	29	7,548 cases; 7,334 controls	Met increased the risk of AD in Caucasian females.
	Ji et al., 2015	23	6,504 cases; 6,636 controls	Lack of association.

## *BDNF* Val66Met Polymorphism and Ethnicity

Meta-analysis, which is a statistical tool for combining the results of different studies investigating the same topic, can provide convincing and reliable evidence relevant to genetic studies with differing results. Several meta-analyses of *BDNF* Val66Met polymorphism studies have demonstrated that the positive association findings of this polymorphism are dependent on ethnicity (**Table 1**). For example, converging evidence suggests that BDNF is implicated in the pathogenesis of bipolar disorder. In 2003, two research groups reported a significant association between the *BDNF* Val66Met polymorphism and bipolar disorder (Neves-Pereira et al., 2002; Sklar et al., 2002). However, most of the other studies cannot replicate this association (Hong et al., 2003a; Nakata et al., 2003). In 2016, due to the lack of reproducibility, Li et al. (2016) performed a systematic meta-analysis of reports evaluating diverse ethnic groups. They found that the *BDNF* Val66Met polymorphism is significantly associated with bipolar disorder in Europeans, but not in Asians.

Brain-derived neurotrophic factor is characterized by survival-promoting activity in various brain neurons, including midbrain dopaminergic variants. Postmortem brain studies have suggested that BDNF is involved in the pathogenesis of Parkinson’s disease (PD) (Joyce et al., 2002; Hong et al., 2003b). Therefore, genetic studies have tested the association of this polymorphism with PD risk but have reported inconsistent results. A meta-analysis of 12 studies showed no association between PD and this *BDNF* polymorphism in all study subjects (Lee and Song, 2014). However, an ethnicity-specific meta-analysis showed that Met carriers have an increased susceptibility to PD in Europeans, but not in Asians.

Strong evidence suggests genetic predisposition to suicidal behaviors (Tsai et al., 2011). To determine the genetic effect of the *BDNF* Val66Met polymorphism on suicidal behaviors, a meta-analysis evaluated 23 studies, including 4,532 patients and 5,364 controls, but found no evidence of an association between this polymorphism and suicidal behaviors (Gonzalez-Castro et al., 2017). However, a significantly increased risk was found in a subgroup analysis by ethnicity in Asian populations (Val homozygotes vs. Met carriers: odds ratio [OR]: 1.36; 95% confidence interval [CI], 1.04–1.78) and in Caucasian populations (Met homozygotes vs. Val carriers: OR: 1.96; 95% CI, 1.58–2.43).

The disparate associations among ethnic groups may be attributed to several reasons. First, considerable *BDNF* allele and haplotype diversity is present among populations globally, and the frequency of the Met allele considerably ranges from 0 to 72% across populations (Petryshen et al., 2010). The low prevalence of the risk allele in some populations may lead to an inadequate population size in studies validating associations found to be significant in low-powered studies. Second, the Met allele is present in different population-specific haplotypes in Caucasians and Asians (Petryshen et al., 2010). If the *BDNF* Val66Met polymorphism is not the true risk variant but links to the probable true functional loci with differing strengths among populations, different associations with the Val66Met polymorphism may be found due to different haplotypic backgrounds. Third, different interactions may occur between the *BDNF* Val66Met polymorphism with other genetic or environmental features that vary among ethnic groups.

## Genetic Model for Analysis of *BDNF* Val66Met Polymorphism

The genetic model for the analysis of an single-nucleotide polymorphism (SNP), such as the *BDNF* Val66Met polymorphism, may be dominant (Met carriers vs. Val/Val), codominant (Met/Met vs. Val/Met vs. Val/Val), or recessive (Met/Met vs. Val carriers). The *BDNF* Met allelic frequency is often reported to be high in Asian populations but low in Caucasian, Central and South American, and African populations (Tsai et al., 2010; Hong et al., 2011; Gonzalez-Castro et al., 2017). Many studies in non-Asian populations have grouped carriers of *BDNF* Val/Met and Met/Met genotypes together as Met carriers because of the small number of Met homozygotes. However, whether the Met allele is dominant, codominant, or recessive is unclear. Furthermore, stratifying the *BDNF* Val66Met polymorphism into two genotypic groups may ignore the molecular heterosis effect. For example, a meta-analysis suggested that Val/Met heterozygotes show higher antidepressant therapeutic effects than Val or Met homozygotes, particularly Asian patients (Verhagen et al., 2010; Zou et al., 2010; Yan et al., 2014). This is referred to as the positive molecular heterosis effect, in which subjects heterozygous for a specific genetic polymorphism show a greater effect (Tsai et al., 2003; Liu et al., 2014). This observation is consistent with the findings an animal study showing that although BDNF exerts an antidepressant effect, very high BDNF expression may have an unfavorable effect on mood (Govindarajan et al., 2006).

The association between the *BDNF* Val66Met polymorphism and panic disorder is inconclusive given the mixed findings (Lam et al., 2004; Chen and Tsai, 2016). A meta-analysis of six studies found no association between the polymorphism and panic disorder in the dominant model (Chen et al., 2017). However, in the recessive model, a significant association was found between the *BDNF* Val66Met polymorphism and panic disorder.

## *BDNF* Val66Met Polymorphism and Sex

There are sex differences in brain BDNF and its receptor expression. Animal study illustrated that male mice have higher BDNF in the frontal cortex, hippocampus and brain stem (Szapacs et al., 2004). The distribution of phosphorylated TrkB receptor in the mouse hippocampal formation depends on sex and estrous cycle stages that phosphorylated TrkB were more abundant in high-estradiol states (proestrus females) than low-estradiol states (estrus and diestrus females and males) (Spencer-Segal et al., 2011). In human, postmortem study found that there is no significant difference in hippocampal BDNF levels between the two genders but female subjects have higher BDNF in the prefrontal cortex (Hayley et al., 2015). Sex differences in the level of BDNF and its receptor in different brain regions could potentially explain some of the disorder-specific sex differences in the association of *BDNF* Val66Met polymorphism.

In the brain, sex hormones and BDNF have mutual effects. The first linkage between BDNF and sex steroids was indicated in a study showing co-localization of BDNF and its receptor in the estrogen receptor (ER) mRNA-containing neurons during forebrain development (Toran-Allerand et al., 1992). Evidence from animal studies suggested that estrogen modulates *BDNF* expressions through at least four different mechanisms (Gibbs, 1998; Chan and Ye, 2017). First of all, estrogen can directly induce *BDNF* expression by activating ER. Second, estrogen modifies the activity of *BDNF* promoter epigenetically. Third, the ER regulates the activity of CREB, a major transcription factor that controls *BDNF* expression in neurons, through non-genomic activities. Lastly, estrogen affects *BDNF* expression indirectly via inter-neuronal activity. In contrast, evidence suggests that some estrogen actions are mediated by BDNF. For example, BDNF was reported to modulate estradiol-induced dendritic spine formation in rat hippocampal neurons (Murphy et al., 1998).

Within the hippocampus, estrogen and BDNF both interact with a number of common receptors, enzymes and proteins such as MAP kinase, ERKs, PI3 kinase, CaMKII, CREB, and Src/Fyn (Luine and Frankfurt, 2013). The interactions between BDNF and estrogen affect hippocampal neurons during development and in adulthood, and these interactions play an important role in the normal brain as well as in diseases (Harte-Hargrove et al., 2013).

When compared with estrogen, the effect of androgen on *BDNF* expression is less studied. Study in mice demonstrated that gonadectomy induced a significant decrease in the BDNF levels in the hippocampal CA1 area, which were prevented by replacement of testosterone, the major component of androgens (Li et al., 2012). Androgens are crucial for the development of male-specific behaviors and for physiological functioning. Animal studies have demonstrated that BDNF and androgens may work cooperatively to influence neuronal plasticity and modulate hippocampal function (Ottem et al., 2013; Atwi et al., 2016).

An animal study demonstrated the effect of sex hormones on BDNF; female *BDNF*^Met/Met^ transgenic mice exhibited significant fluctuations in anxiety-like behaviors over the estrous cycle; specifically, these mice exhibited increased anxiety-like behaviors during the estrus phase (Bath et al., 2012a). A human study found that during the menstrual cycle, plasma BDNF levels were significantly higher in the luteal phase than in the follicular phase (Begliuomini et al., 2007). A recent multimodal imaging study in 39 healthy women found an ovarian hormone-by-*BDNF* interaction on working memory-related hippocampal function, suggesting that differential hippocampal recruitment occurs in Met carriers but only in the presence of estradiol (Wei et al., 2017).

Studies from the fields of genetic epidemiology, clinical psychiatry, behavioral neuroscience and neuroimaging suggest that the *BDNF* Val66Met polymorphism may not be a major risk allele for the development of schizophrenia *per se*, but the polymorphism modulates a range of clinical features of the illness, including age of onset, symptoms, therapeutic responsiveness, neurocognitive function and brain morphology (Notaras et al., 2015a). Findings from clinical and animal studies of schizophrenia showed that estrogen may provide a protective effect in schizophrenia, including through mediating *BDNF* expression and activity (Wu et al., 2013). This posited estrogen-BDNF interaction could play a key role in sex differences in clinical aspects of schizophrenia.

Because sex hormones may affect BDNF function, sex may contribute to the discrepancy in the findings of *BDNF* Val66Met genetic studies. For example, BDNF plays a critical role in neuronal survival, synaptic plasticity, and memory (Tsai, 2003b; Huang et al., 2014; Lin et al., 2016). Therefore, *BDNF* is a favorable candidate for Alzheimer’s disease (AD) genetic studies. The first genetic association study of the *BDNF* Val66Met polymorphism and AD demonstrated that Val is the risk allele for AD (Ventriglia et al., 2002). Studies attempting to replicate this finding have obtained inconsistent results (Tsai et al., 2004a, 2006). To establish the true effect of the *BDNF* polymorphism on AD, Fukumoto et al. (2010) performed a meta-analysis of studies investigating the effects of the *BDNF* Val66Met polymorphism on AD. The results revealed a clear sex difference in the allelic association; the Met allele confers susceptibility to AD in women (*P* = 0.002), but not in men. This finding suggests that the *BDNF* Val66Met polymorphism has a sexually dimorphic effect on susceptibility to AD. This result is consistent with the finding that the *BDNF* Val66Met polymorphism has a sex-specific role (in women, but not in men) in cognitive function during normal cognitive aging (Laing et al., 2012). Similarly, a meta-analysis of studies evaluating the effect of the *BDNF* Val66Met polymorphism on major depressive disorder showed that, in the total sample, the *BDNF* Val66Met polymorphism is not significantly associated with depression; however, sex-stratified allelic and genotypic analyses revealed significant effects in men (Verhagen et al., 2010).

Sex-specific associations of the *BDNF* Val66Met polymorphism with cortisol responses to mental stress (Jiang et al., 2017), neurocognitive function in schizophrenia (Kim et al., 2016), sympathetic tone (Chang et al., 2014), HPA axis reactivity to psychological stress (Shalev et al., 2009), and attention-deficit/hyperactivity disorder (ADHD) (Cho et al., 2010) have also been reported.

In addition to *BDNF* Val66Met genetic studies in neuropsychiatric diseases, studies of serum BDNF levels in neuropsychiatric diseases have shown a sex effect. For example, BDNF has been implicated in the pathogenesis of ADHD (Tsai, 2003a, 2017a; Tzang et al., 2013). In a recent meta-analysis of studies examining peripheral BDNF levels in ADHD, although no significant difference was found in peripheral BDNF levels between ADHD patients and normal controls, overall, BDNF levels were significantly higher in male ADHD subjects than in male controls (Zhang et al., 2017).

## *BDNF* Val66Met Polymorphism and Age

The tissue expression of BDNF varies across the life span. The human serum BDNF concentration increases in the first several years of life and then slightly decreases in adulthood (Katoh-Semba et al., 2007). Another study found that plasma BDNF levels decrease significantly with age, whereas platelet levels do not, suggesting the age effect on BDNF levels is tissue-specific (Lommatzsch et al., 2005). Age not only affects BDNF expression but also affects the conversion of proBDNF to mature BDNF. A study examining BDNF expression in mouse hippocampal lysates showed that the expression of both pro- and mature BDNF was low on postnatal day 0 (Yang et al., 2014). The expression of proBDNF peaked on postnatal day 15 and declined in later stages. The expression of mature BDNF peaked on postnatal day 21 and plateaued in adulthood (Yang et al., 2014).

Brain-derived neurotrophic factor is involved in pruning and shaping the adolescent brain and has been implicated in the pathogenesis of neurodevelopmental disorders. Study in male mice found significant changes in BDNF expressions in the forebrain regions during weeks 7–10 (Hill et al., 2012). Castration and testosterone replacement experiments demonstrated an androgen receptor-dependent effect on BDNF-TrkB signaling in the forebrain and hippocampal regions during adolescence. Female mice showed changes in BDNF-TrkB signaling at a much earlier time point (weeks 4–8) in the forebrain and hippocampal regions (Hill et al., 2012). During adolescence, the incidence of mental illnesses such as schizophrenia and depression increases substantially. Accordingly, altered synthesis and/or activity of BDNF, which are key regulators of many mental disorders, may contribute to the development of these mental diseases in adolescence.

Studies examining the (mRNA and protein) expression of BDNF and its receptors in the hippocampus and hypothalamus throughout the life span of rats have found that receptors, rather than BDNF itself, are impaired with aging (Silhol et al., 2005; Rage et al., 2007). These findings suggest that age also affects BDNF signaling through changes in its receptor.

Based on the aforementioned findings, age may mediate the effect of the *BDNF* Val66Met polymorphism on disease susceptibility. In our studies of the *BDNF* Val66Met polymorphism and major depression, we found that Met carriers have an increased risk of geriatric depression, but not non-geriatric depression (Hong et al., 2003a; Tsai et al., 2003; Hwang et al., 2006). This finding was further confirmed by a meta-analysis of five studies including 523 patients with geriatric depression and 1,220 psychiatrically healthy controls (Pei et al., 2012). Similarly, a recent study showed a complex relationship between the *BDNF* Val66Met polymorphism and mortality for traumatic brain injury, and that study demonstrated that this polymorphism interacts with age to influence survival predictions beyond clinical variables alone (Failla et al., 2015).

## *BDNF* Val66Met Polymorphism and Gene–Gene Interaction

Brain-derived neurotrophic factor exerts its trophic action mainly by signaling through the trkB receptor (encoded by the *NTRK2* gene). The trkB signaling pathway involves many proteins that also possibly affect BDNF function. In addition, the proteolytic cleavage of proBDNF (a BDNF precursor with effects opposite to those of BDNF) to BDNF by plasmin determines the direction of BDNF action (Lu et al., 2005; Tsai, 2017b). Therefore, polymorphisms in the genes encoding proteins involved in the trkB or plasmin signaling pathway may interact with the *BDNF* Val66Met polymorphism to affect disease susceptibility (Tsai, 2004a, 2007b; Hwang et al., 2006). For example, using a generalized multifactor dimensionality reduction method, we found the *BDNF* Val66Met polymorphism interacts with *NTRK2* genetic polymorphisms (rs1187323 and rs1778929) to affect susceptibility to geriatric depression (Lin et al., 2009).

The *BDNF* Val66Met polymorphism has also been reported to interact with the 𝜀4 allele of *apolipoprotein E* (*APOE*), thereby affecting AD susceptibility in women (Zhao Q. et al., 2017). Another study found that the *BDNF* Val66Met polymorphism interacts with the serotonin transporter gene polymorphism to influence neuroticism-related personality traits (Terracciano et al., 2010). Recently, Prats et al. (2017) demonstrated an interaction between the rs1475157 polymorphism of *NRN1* (a neurotrophic factor involved in synaptic plasticity) and the *BDNF* Val66Met polymorphism; this interaction modulated depressive symptoms in 410 non-clinical participants (Prats et al., 2017).

To analyze interactions in genetic data, many statistical methods have been suggested, with most of them relying on statistical regression models. Given the known limitations of classical methods, approaches with the machine-learning have also become favorable. Among them, the multifactor dimensionality reduction (MDR), a powerful statistical tool for detecting and modeling epistasis, has been widely applied (Ritchie et al., 2001). Polygenic risk score is another approach to summarize the additive trait variance captured by a set of genetic markers that do not individually achieve significance in a large-scale association study (Baker et al., 2018).

## Interaction Between *BDNF* Val66Met Polymorphism and Environmental Factors

Evidence suggests that interactions between genes and the environment influence brain development and the risk of neuropsychiatric diseases (Keverne, 2014; Booij et al., 2015; Lin et al., 2017; Misiak et al., 2017). Many environmental factors (such as prenatal adverse environments, childhood trauma, weather and life stress) have been found to play an important role in the causality of brain diseases.

The *BDNF* Val66Met polymorphism has been reported to interact with early life stress; thus, Val carriers with childhood trauma are more susceptible to the occurrence of subclinical psychotic experiences (de Castro-Catala et al., 2016). Another study in subjects with the schizophrenia spectrum or bipolar disorder demonstrated that Met carriers with high levels of childhood trauma have significantly low levels of blood *BDNF* mRNA and decreased CA2/3 and CA4 subfield areas in the dentate gyrus (Aas et al., 2014).

The *BDNF* Val66Met polymorphism has been long considered an important candidate for reducing depression risk; however, inconsistent findings have been obtained. A meta-analysis with a pooled total of 14,233 participants found that the Met allele significantly moderates the link between life stress and depression risk (Hosang et al., 2014). When stratified by the type of environmental stressor, the interaction between the *BDNF* Val66Met polymorphism and life stress in depression became stronger for stressful life events rather than for childhood adversity. The findings were replicated by a recent meta-analysis of 31 studies, involving of 21,060 participants, providing further evidence for an interaction between the *BDNF* Val66Met polymorphism and life stress in depression (Zhao M. et al., 2017).

Epigenetic studies have suggested that histone modifications, DNA methylation, and hydroxymethylation are possible mediators linking individual response to environmental factors and brain diseases (McEwen et al., 2015). These mediators may change the pattern of gene expression, influencing protein levels and ultimately shaping phenotypes during the life span. A study evaluating *BDNF* Val66Met polymorphism methylation in the peripheral blood of healthy subjects demonstrated that the increased methylation was associated with hypoxia-related early life events and impaired working memory in Val/Val individuals, and the opposite was true for Val/Met individuals (Ursini et al., 2016).

The interplay of genetic, epigenetic, and environmental factors may influence cognitive function. A study in normal subjects and subjects with amnestic mild cognitive impairment (aMCI) demonstrated that the increased *BDNF* promoter methylation status was associated with aMCI and its progression to AD (Xie et al., 2017). The interaction between DNA methylation and Met homozygosity increased the risk of aMCI and its progression to AD.

An epigenetic study of anxiety/depression in older women found higher *BDNF* DNA methylation in subjects with anxiety/depression than in controls, and this difference was more pronounced in *BDNF* Val66Met heterozygotes than in Val homozygotes (Chagnon et al., 2015).

It should be noted that, in terms of the two-hit hypothesis, there are studies which show that a second hit actually led to improvements, and some genetic polymorphisms, including *BDNF* Val66Met polymorphism, may actually increase resilience. For example, a recent study showed that *BDNF*^Met/Met^ transgenic mice had spatial and fear-associated memory deficits, but corticosterone treatment recovered this phenotype (Notaras et al., 2017).

## *BDNF*^Met/Met^ Transgenic Mice

Chen et al. (2006) generated an inbred genetic knock-in mouse (*BDNF*^Met/Met^) that recapitulates the phenotypic hallmarks of human carriers with the Met allele. *BDNF*^Met/Met^ mice represent a potential model to study the biological mechanism of this polymorphism in the brain.

*BDNF*^Met/Met^ mice had decreased basal BDNF protein levels in the hippocampus, which could not be normalized by antidepressant (fluoxetine) administration (Bath et al., 2012b). *BDNF*^Met/Met^ mice also showed impaired survival of newly generated cells and LTP in the dentate gyrus (Bath et al., 2012b). A recent study demonstrated that *BDNF*^Met/Met^ mice exhibited diminished development of serotonergic fibers projecting particularly to the prefrontal cortex compared with wild-type mice; this diminished development was rescued by fluoxetine administration during peri-adolescence (Dincheva et al., 2017).

Compared with wild-type mice, significant decreases of 13.7% ± 0.7% and 14.4% ± 0.7% were observed in the hippocampal volume of *BDNF*^+/Met^ and *BDNF*^Met/Met^ mice, respectively (Chen et al., 2006). The transgenic mice showed increased depression and anxiety-like behaviors in stressful settings, and the behaviors were not normalized by antidepressant (fluoxetine) administration (Chen et al., 2006; Yu et al., 2012). In addition, the variant mice showed impaired learning of cues that signal safety (Soliman et al., 2010). These findings provide an example of a human genetic variant that has been modeled in transgenic mice can produce similar phenotypic hallmarks observed in some clinical studies.

The aforementioned findings should be interpreted with caution because not all findings demonstrated in *BDNF*^Met/Met^ mice have been consistently found in human studies. For example, *BDNF*^Met/Met^ mice had a decreased hippocampal volume compared with that of wild-type mice (Chen et al., 2006). An earlier report also showed that human Met carriers had reduced hippocampal gray matter volume compared with that of Val homozygotes (Pezawas et al., 2004). However, following imaging genetic studies have shown controversial results regarding the genetic effect of *BDNF* Val66Met on hippocampal volumes in normal subjects (Harrisberger et al., 2014; Liu et al., 2014). A meta-analysis including 5,298 healthy subjects revealed no significant *BDNF* genotype effect on hippocampal volume (Harrisberger et al., 2014).

Another example is the genetic association studies of the *BDNF* Val66Met polymorphism and cognitive function, which has been the focus of several clinical studies. Cognitive impairment has been reported in a mouse model of the *BDNF* Met allele (Chen et al., 2006; Dincheva et al., 2012). Conflicting findings have been obtained for the genetic effect of *BDNF* Val66Met on human cognitive function (Tsai et al., 2004b, 2008a; Hong et al., 2011). A meta-analysis including 7,095 individuals failed to support significant genetic associations between the Val66Met polymorphism and any of the cognitive phenotypes (Mandelman and Grigorenko, 2012).

Brain-derived neurotrophic factor has been implicated in the pathogenesis of major depression (Duman et al., 1997; Tsai et al., 2008b). Animal studies have demonstrated that *BDNF*^Met/Met^ mice exhibited depression-like behaviors in stressful situations (Chen et al., 2006; Yu et al., 2012). However, in clinical studies, we found the Met allele is not associated with depression in either psychiatric outpatients or inpatients (Hong et al., 2003a; Tsai et al., 2003).

Finally, it should be noted that the knock-in mouse model developed by the Lee group simply replaced the valine (which in rodents is in position 68, not 66) with a methionine (Chen et al., 2006). Recently the Ron research team generated another transgenic mice carrying the mouse homolog of the human *BDNF* Met allele (Met68*BDNF*) (Warnault et al., 2016). Using this model, they demonstrated that Met allele increases the risk of compulsive alcohol drinking which can be reversed by directly activating the TrkB receptor (Warnault et al., 2016).

It is not known if and how this slight difference with the human *BDNF* Val66Met polymorphism affects the validity of these mouse models. A more precise transgenic model was developed by the Gogos group, where the mice were ‘humanized’ by inserting a small stretch of human sequence, including Val/Met at position 66 (Cao et al., 2007). This genetic manipulation generated knock-in alleles that express human *BDNF* genes controlled by endogenous mouse *Bdnf* regulatory elements. This one has now been used by several other investigators. For example, recent studies using this *hBDNF*^V al66Met^ knock-in mice, van den Buuse et al. (2017) showed that the *BDNF* Val66Met Val/Met and Met/Met genotypes are more sensitive than the Val/Val genotype to the effect of apomorphine on prepulse inhibition. A history of stress, modeled by long-term treatment with corticosterone in young adults, increases the effects of apomorphine in Val/Val mice (van den Buuse et al., 2017).

## Findings of the Novel Function of *BDNF* Val66Met Polymorphism

The first study investigating the function of this polymorphism demonstrated that *BDNF* Val66Met polymorphism affects activity-dependent BDNF release (Egan et al., 2003). In addition to this genetic effect, recent studies have found more functional effects for this polymorphism.

Brain-derived neurotrophic factor is initially synthesized as the precursor protein proBDNF, which is then cleaved by intracellular (furin/PC1) or extracellular peptidase enzymes (tPA/plasmin/MMP) into bioactive mature BDNF and pro-peptide (or pro-domain) (Pang et al., 2004). The Val66Met substitution is present in the BDNF pro-peptide region. The BDNF pro-peptide is detected in the hippocampus, and the application of the Met-type, but not Val-type, BDNF pro-peptide can induce acute growth cone retraction, suggesting that the Met-type pro-peptide is a new active ligand that can modulate neuronal morphology (Anastasia et al., 2013).

The BDNF pro-peptide functions as a modulator of synaptic plasticity by enhancing hippocampal long-term depression (LTD) (Mizui et al., 2015). Mizui et al. found that the Val-type BDNF pro-peptide facilitates low-frequency stimulation–induced hippocampal LTD, whereas the Met-type pro-peptide attenuates LTD (Mizui et al., 2015).

The BDNF pro-peptide can bind to mature BDNF with high affinity, and compared with the complex with the Val-type pro-peptide, the complex with the Met-type pro-peptide is more stable, suggesting that the *BDNF* Val66Met polymorphism affects the stability of the complex formed between BDNF and its pro-peptide (Uegaki et al., 2017).

The *BDNF* Val66Met polymorphism may affect the protein or mRNA expression of BDNF. The effect of the Val66Met polymorphism on the constitutive expression of BDNF was tested in HEK293T cells transiently transfected with recombinant plasmids to induce overexpression of either the Val or Met variant (Jin et al., 2015). A significant decrease in secreted BDNF protein levels in the culture supernatants of cells overexpressing the Met variant was found. In the same study, Met carriers had increased blood *BDNF* mRNA and protein levels. A higher circulating BDNF concentration associated with the Met allele was also found in a large cohort (Kaess et al., 2015), but a negative association was also found (Jiang et al., 2009). In a meta-analysis, no association was found between serum BDNF levels and the Val66Met polymorphism (Terracciano et al., 2013).

## Is Met or Val the Risk Allele?

The *BDNF* Val66Met polymorphism has been reported to be associated with psychiatric disorders, including obsessive-compulsive disorder, schizophrenia, psychosis, major depression, anxiety, and eating disorders (Hong et al., 2011; Notaras et al., 2015b). Most positive association studies have reported that the Met allele is the risk allele for psychiatric disease given that Met carriers exhibit reduced activity-dependent secretion of BDNF (**Table 1**). However, the higher activity *BDNF* Val allele is associated with bipolar disorder (Neves-Pereira et al., 2002; Sklar et al., 2002) and substance use disorder (Cheng et al., 2005; Liu et al., 2005; Sim et al., 2010). In the Mexican–American population, it has been found that individuals homozygous for the Val allele have an increased chance of depression (Ribeiro et al., 2007). These findings suggest that this *BDNF* polymorphism has pleiotropic effects on multiple phenotypes; thus, this polymorphism imparts separate advantageous traits and disadvantageous traits in the same organism.

The different effects of this polymorphism in different disorders here could be due to the differential expression of BDNF and its receptor in different regions of the brain. For example, over or under activity-dependent secretion of BDNF will have varying effects on amygdala related behaviors (e.g., fear/anxiety) when compared with cognition (hippocampal-dependent) (Andero et al., 2014; Ilchibaeva et al., 2018).

Furthermore, evidence suggests that increased BDNF activity has a deleterious effect and may be implicated in the pathogenesis of some diseases (Tsai, 2005, 2006, 2007a,c). For example, increased BDNF activity in the ventral tegmental area-nucleus accumbens (VTA-NAc) pathway may be implicated in the pathogenesis of major depression (Eisch et al., 2003). Evidence also suggests that BDNF overactivity in the brain may be implicated in the pathogenesis of bipolar disorder (Tsai, 2004b), substance abuse (Tsai, 2007a), and autism (Tsai, 2005). Moreover, the genetic overexpression of the BDNF mature isoform in female mice impaired working memory functions, reduced breeding efficiency, increased anxiety-like behaviors, impaired prepulse inhibition, and elicited higher susceptibility to seizures (Govindarajan et al., 2006; Papaleo et al., 2011). Thus, the Val allele, which is associated with the increased activity-dependent secretion of BDNF, may be the risk allele for some neuropsychiatric diseases.

## Other *BDNF* Polymorphisms

Investigating a single *BDNF* polymorphism (i.e., the Val66Met polymorphism) might only reveal some of the *BDNF* genetic variability and result in the overlooking of some information from other *BDNF* SNPs (Tsai et al., 2010; Yeh et al., 2015). Furthermore, the use of a haplotype constructed by several tag *BDNF* SNPs can improve genotyping efficiency by reducing the number of polymorphisms to be genotyped, and the haplotype itself may also tag other genetic variants that affect gene function.

Genetic studies of other *BDNF* polymorphisms have been conducted. For example, Proschel et al. (1992) identified a dinucleotide repeat polymorphism (GT) that maps 1,040 bp upstream from the transcription start site (Proschel et al., 1992). The *BDNF* GT repeat polymorphism is associated with age at onset, therapeutic response, susceptibility, and chlorpromazine-induced extrapyramidal syndrome in schizophrenia (Krebs et al., 2000; Xu et al., 2008).

Another common *BDNF* SNP, namely the C270T polymorphism (rs56164415) in the *BDNF* 5′ non-coding region, has been identified and reported to be associated with AD (Kunugi et al., 2001).

By sequencing the entire *BDNF* gene and the 5-kb flanking region, Licinio et al. (2009) demonstrated that six *BDNF* SNPs (rs12273539, rs11030103, rs6265, rs28722151, rs41282918, and rs11030101) are significantly associated with MDD.

## Conclusion

Considering the important role of BDNF in the brain and the functional effect of the common *BDNF* Val66Met polymorphism, this polymorphism is one of the most studied polymorphisms in neuropsychiatric diseases. However, following studies have been unable to replicate most positive findings in initial genetic studies. In this review, we highlighted critical issues in *BDNF* Val66Met studies, which may affect the findings of these studies. Most neuropsychiatric diseases are complex diseases that are dependent on many genetic and environmental factors that cannot be analyzed by conventional genetic association studies. Future studies should analyze various *BDNF* polymorphisms and these related factors by using machine learning techniques to accurately understand the genetic effect of *BDNF* on disease pathogenesis.

## Author Contributions

The author confirms being the sole contributor of this work and approved it for publication.

## Conflict of Interest Statement

The author declares that the research was conducted in the absence of any commercial or financial relationships that could be construed as a potential conflict of interest.
